# Mechanism of Synergistic Action of Isochlorogenic Acid C and Ciprofloxacin Against Drug-Resistant *Escherichia coli*

**DOI:** 10.3390/biom16091328

**Published:** 2026-09-12

**Authors:** Yubin Bai, Xu Chen, Rongbin Hu, Xun Gao, Xiaojuan Wei, Zixuan Shang, Jiyu Zhang, Zhen Zhu, Mingze Cao, Bing Li

**Affiliations:** 1 Key Laboratory of New Animal Drug Project of Gansu Province, Key Laboratory of Veterinary Pharmaceutical Development of the Ministry of Agriculture and Rural Affairs, Lanzhou Institute of Husbandry and Pharmaceutical Sciences of CAAS, Lanzhou 730050, China; baiyubin@caas.cn (Y.B.); chenxu000310@126.com (X.C.); hurongbin@caas.cn (R.H.); gaoxun@caas.cn (X.G.); weixiaojuan@caas.cn (X.W.); szxuanszxuan@163.com (Z.S.); zhangjiyu@caas.cn (J.Z.); 2 School of Life Science and Food Engineering, Hebei University of Engineering, Handan 056038, China; zhuzhen234@yeah.net

**Keywords:** ICAC, Ciprofloxacin, *Escherichia coli*, synergistic effect, mechanism

## Abstract

Background: The rapid emergence of antibiotic resistance in *E. coli* has outpaced the development of new drugs, creating an urgent demand for novel therapeutic strategies. Methods: We screened ten antibiotics in combination with isochlorogenic acid C (ICAC) against multidrug-resistant (MDR) *E. coli*. Synergistic effects of antibiotics and ICAC were evaluated by measuring the leakage of alkaline phosphatase (AKP), potassium ions (K^+^), as well as changes in total protein, and ATP levels. The integrity of bacterial membrane and cell wall was visualized by SEM and CLSM. The underlying mechanisms were elucidated via proteomic analysis and molecular docking. Finally, the protective effect of ICAC in vivo was evaluated using an intraperitoneal infection model in SPF female BALB/c mice. Results: ICAC exerted a specific synergistic effect with ciprofloxacin, significantly enhancing its efficacy. The combination disrupted bacterial membrane and cell wall integrity, increased permeability, and inhibited ATP synthesis. Mechanistically, ICAC may target OmpF and inhibit fatty acid biosynthesis. This interference reduced β-oxidation and the influx of acetyl-CoA into the TCA cycle, thereby impairing bacterial energy metabolism and destabilizing the membrane. In vivo validation using a mouse peritonitis model confirmed that the combination treatment effectively alleviated systemic *E. coli* infection. Conclusion: ICAC may potentiate the antibacterial activity of ciprofloxacin through targeting of OmpF, which disrupts bacterial membrane integrity and energy metabolism, providing a promising candidate strategy for combating infections caused by the tested antibiotic-resistant *E. coli*.

## 1. Introduction

Pathogenic *Escherichia coli* (*E. coli*) is a common Gram-negative bacterial pathogen, which can cause diarrhea, urinary tract infections, sepsis and other diseases [1,2]. However, the excessive use of antibiotics in both animals and humans has driven the rise in multidrug-resistant (MDR) *E. coli* in recent decades [3,4]. Consequently, with the emergence of MDR *E. coli*, the therapeutic efficacy of antibiotics has diminished. Moreover, the development of novel antibiotics has failed to keep pace with the rapid dissemination and evolution of bacterial resistance, posing a formidable clinical challenge [5]. According to the World Health Organization (WHO), drug-resistant bacterial infections could claim millions of lives annually by 2050, imposing a substantial economic burden [6,7]. Therefore, there is an urgent need to develop novel antibacterial strategies to address this crisis.

Natural compounds represent a rich reservoir of antibacterial agents, and demonstrate efficacy either as standalone treatments or in combination with conventional antimicrobials [8]. Phenolic acids constitute a class of secondary metabolites utilized in the treatment of bacterial infections [9]. Studies have demonstrated that phenolic acids exhibit antibacterial activity against both Gram-positive and Gram-negative bacteria [10,11]. Previous studies have reported that the combination of phenolic acids and antibiotics inhibits the growth of *E. coli* by enhancing the permeability of bacterial cell membrane and disrupting bacterial cell walls [12].The antioxidant, anti-inflammatory and cardiovascular protective properties of ICAC have been extensively investigated [13], yet its specific antibacterial effects and the mechanisms underlying its combined action with antibiotics remain unexplored.

This study evaluated the in vitro antimicrobial activity of ICAC in combination with various antibiotics, including ciprofloxacin, tetracycline, meropenem and ceftriaxone, using minimum inhibitory concentration (MIC) assay, synergy tests, and time–kill assays. Furthermore, the underlying mechanisms were elucidated by assessing the permeability and structural integrity of both bacterial cell walls and membranes. These findings provide a solid theoretical foundation for utilizing natural compounds to curb the emergence and spread of bacterial resistance.

## 2. Materials and Methods

### 2.1. Materials and Bacterial Strains

*E. coli* ATCC 25922 was obtained from the American Type Culture Collection (ATCC). *E. coli* XJ-24 was isolated and preserved at the Lanzhou Institute of Husbandry and Pharmaceutical Sciences, Chinese Academy of Agricultural Sciences. All strains were cultured in Mueller Hinton Broth (MHB, Guangdong Huankai Microbial Sci. & Tech. Co., Ltd., Guangzhou, Guangdong, China) and Mueller Hinton Agar (MHA, Guangdong Huankai Microbial Sci. & Tech. Co., Ltd., Guangzhou, Guangdong, China). Ceftriaxone sodium, polymyxin, tigecycline, meropenem, amikacin, kanamycin, aztreonam, tetracycline, ciprofloxacin, and amoxicillin were purchased from Beijing Solarbio Science & Technology Co., Ltd., (Beijing, China). ICAC was purchased from Chenguang Biotechnology Group Co., Ltd., (Handan, China). All antibiotic stock solutions were prepared in sterile distilled water.

### 2.2. Minimum Inhibitory Concentration (MIC) Assay

The MICs of the compounds used in this study were determined using broth microdilution assay [14]. Briefly, bacterial strains were cultured in MHB medium at 37 °C. Subsequently, the bacterial cultures were resuspended to a cell density equivalent to the 0.5 McFarland standard and further diluted 100-fold with MHB. For antibacterial susceptibility testing, ICAC and various antibiotics were serially two-fold diluted and added to 96-well microplates. Each well was then incubated with 100 µL of the diluted bacterial suspension, and the plates were incubated for 16–18 h at 37 °C. Sterility and blank growth control groups were set up simultaneously in each independent experiment. The MIC value was defined as the minimum compound concentration that completely suppressed visible bacterial growth.

### 2.3. Antibiotic Synergism Tests

The interaction between ICAC and antibiotics in combination was determined using a checkerboard assay based on the fractional inhibitory concentration index (FICI) [15]. Briefly, antibiotics and ICAC were prepared in the same manner as for the MIC assay. Serial two-fold dilutions of ICAC were combined with serial two-fold dilutions of antibiotics, followed by incubation at 37 °C for 18 h. The FICI was used to evaluate the combined inhibitory effect using the following equation:

FICI = (MIC A in combination/MIC A alone) + (MIC B in combination/MIC B alone)(1)

where MIC A represents the MIC of the ICAC, and MIC B represents the MIC of the antibiotics. Interactions between the two agents were classified as synergism, no interaction, and antagonism when the FICI value is ≤0.5, >0.5–4.0, and >4.0, respectively.

### 2.4. Time–Kill Assay

The time–kill assay was used to evaluate the synergistic bactericidal effect of ICAC in combination with ciprofloxacin [16]. *E. coli* ATCC 25922 and XJ-24 in the exponential phase were diluted to 1 × 10^6^ CFU/mL in MHB and then treated with 1/2 MIC ICAC, 1/2 MIC ciprofloxacin and the combination of 1/2 MIC ICAC plus 1/2 MIC ciprofloxacin. Bacterial suspensions without drug treatment were set as the control group. At the time points (0, 4, 8, 12 and 24 h), 100 µL of bacterial suspension was serially diluted with MHB, spread onto MHA plates, and incubated overnight at 37 °C. Colony counts were subsequently determined. 

### 2.5. Cell Wall Permeability

Alkaline phosphatase (AKP) naturally resides in the periplasmic space of Gram-negative bacteria. Intact bacterial cell walls serve as a physical barrier that blocks AKP leakage into the extracellular environment. Once the cell wall structure is damaged and membrane permeability increases, intracellular AKP is released from the periplasmic space. Therefore, extracellular AKP activity was determined to evaluate cell wall permeability in this study. All experimental procedures were conducted using a commercial AKP assay kit (Nanjing Jiancheng Biotechnology Institute, Nanjing, China). Bacterial cultures of *E. coli* ATCC 25922 and XJ-24 in the logarithmic growth phase were centrifuged for 3 min, and the resulting cell pellets were resuspended in PBS. Subsequently, bacterial suspensions were treated with 1/2 MIC ICAC, 1/2 MIC ciprofloxacin or the combination of 1/2 MIC ICAC plus 1/2 MIC ciprofloxacin. Untreated bacterial suspensions served as the control group. After 6 h of incubation at 37 °C, all samples were centrifuged at 12,000 rpm, and the supernatant was collected for analysis. Separately, 5 μL of sterile distilled water, 0.1 mg/mL phenol standard solution, and prepared sample supernatant were added to 96-well plates. Each well was supplemented with 50 μL of buffer solution and matrix solution, followed by thorough mixing. After incubation at 37 °C for 15 min, 150 μL of chromogenic substrate was added to each well. The plates were thoroughly mixed, and the optical density (OD) value was measured at 520 nm according to the manufacturer’s instructions for the AKP assay kit [17].

### 2.6. K^+^ and Protein Leakage Assay

Intact bacterial inner membranes retain cytoplasmic K^+^ and proteins. Damage to the inner membrane compromises its barrier function and increases permeability, resulting in the release of intracellular K^+^ and soluble proteins into the culture supernatant. Therefore, measurement of extracellular K^+^ and protein concentrations allows assessment of inner membrane permeability of *E. coli* following exposure to ICAC and ciprofloxacin. K^+^ and protein leakage assays were performed to investigate inner membrane permeability changes in *E. coli* upon treatment with ciprofloxacin and ICAC [18]. *E. coli* ATCC 25922 and XJ-24 were cultured in MHB at 37 °C for 16 h, then treated with ciprofloxacin and ICAC at 1/2 MIC for 6 h, followed by centrifugation at 11200× *g* for 3 min. Untreated bacterial suspensions served as the control group. Extracellular K^+^ levels were quantified following the manufacturer’s protocol for the Biochemical Assay Reagent (Elabscience, Wuhan, Hubei, China). Protein concentrations were determined using a BCA protein assay kit (meilunbio, Dalian, China).

### 2.7. ATP Assay

Intracellular ATP serves as a key indicator of bacterial energy metabolism and membrane integrity. Damage to the inner membrane increases membrane permeability, leading to leakage of intracellular ATP into the extracellular space. Therefore, intracellular ATP levels were quantified to assess the effects of ICAC and ciprofloxacin, either alone or in combination, on *E. coli*. Changes in intracellular ATP concentration following ciprofloxacin or ICAC treatment were determined using an ATP Assay Kit (Beyotime Biotechnology Co., Ltd., Shanghai, China) [19]. Briefly, *E. coli* ATCC 25922 and XJ-24 in the logarithmic phase were treated with 1/2 MIC ICAC, 1/2 MIC ciprofloxacin, and the combination of both. Untreated bacterial suspensions served as the control group. The samples were incubated at 37 °C for 6 h, centrifuged at 12,000 rpm for 3 min, washed, and resuspended in 0.1 M PBS to a final concentration of 1 × 10^6^ CFU/mL. After another centrifugation step, the supernatants were discarded. According to the manufacturer’s protocol of the ATP Assay Kit, a multifunctional microplate reader was used to evaluate the effects of ciprofloxacin and ICAC on ATP production in *E. coli*.

### 2.8. Measurement of IM Integrity by Automatic Microplate

Propidium iodide (PI) is a nucleic-acid-binding fluorescent dye. Intact bacterial inner membranes can block PI penetration, resulting in negligible background fluorescence. Once the inner membrane is damaged, PI penetrates the impaired membrane and binds to intracellular bacterial DNA. Inner membrane integrity was evaluated using a previously reported method [20]. Briefly, *E. coli* ATCC 25922 and XJ-24 were cultured to logarithmic growth phase and harvested by centrifugation at 12,000 rpm for 3 min. The collected bacterial cells were resuspended in 0.1 M PBS and adjusted to 1 × 10^6^ CFU/mL. Bacterial suspensions were stained with 5 µg/mL PI at 37 °C for 15 min in the dark. Fluorescence intensity was measured at an excitation wavelength of 535 nm and an emission wavelength of 620 nm, with readings recorded every 2 min for 15 min. Subsequently, 1/2 MIC ICAC, 1/2 MIC ciprofloxacin, and the combination of both were rapidly added to the bacterial suspensions. Untreated bacterial suspensions served as the control group. The fluorescence intensity was continuously measured every 2 min for 1 h post-treatment.

### 2.9. Confocal Laser Scanning Microscopy (CLSM)

Inner membrane damage was visualized and analyzed using confocal laser scanning microscopy (CLSM) according to a previously described method [21]. Briefly, *E. coli* ATCC 25922 and XJ-24 in logarithmic growth phase were cultured in LB medium at 37 °C and adjusted to a turbidity equivalent to the 0.5 McFarland standard. The standardized bacterial suspensions were treated with 1/2 MIC ICAC, 1/2 MIC ciprofloxacin, and the combination of both, followed by incubation at 37 °C for 6 h. Untreated bacterial suspensions served as the control group. After incubation, the bacteria cells were centrifuged at 12,000 rpm for 3 min and resuspended in PBS. Subsequently, the cells were stained with cFDA (100 μM) and PI (15 μM), and incubated in the dark for 30 min at 37 °C. Following three washes with PBS to remove unbound dye, the bacterial samples were observed and imaged using a CLSM system (LSM800, Zeiss, Jena, Germany). 

### 2.10. Scanning Electron Microscopy

This experiment was performed with minor modifications according to the method described by Pan et al. [22]. Briefly, bacterial suspensions treated with ciprofloxacin or ICAC were cultured at 37 °C with shaking at 180 rpm, followed by centrifugation at 12,000 rpm for 3 min. Untreated bacterial suspensions served as the blank control group. The cell pellets were rinsed three times with PBS and mixed with 2.5% (*v*/*v*) glutaraldehyde at 4 °C for 12 h. After two additional PBS washes, the bacterial samples were dehydrated using a graded series of ethanol concentrations (30, 50, 70, 90, and 100%) and soaked in tert-butanol for 20 min. Finally, the processed bacterial samples were observed and imaged by SEM (JSM-7500F, JEOL Ltd., Tokyo, Japan).

### 2.11. DIA Quantitative Proteomic

Changes in protein expression of the *E. coli* XJ-24 strain were determined following treatment with ICAC and ciprofloxacin. Bacterial samples were harvested after 6 h of drug incubation, and three biologically independent protein replicates from each experimental condition were used for proteomic sequencing. In brief, total bacterial protein was extracted, and protein concentration was assessed using the Bradford protein quantification kit. Following trypsin digestion, liquid chromatography separation was performed via the Vanquish™ Neo UHPLC system, followed by data-independent acquisition (DIA) mass spectrometry analysis. Post-sequencing data annotation and functional analysis were conducted using InterProScan software (https://www.ebi.ac.uk/interpro/result/InterProScan/iprscan5-R20250208-075705-0178-93699187-p1m/internal-1739001403063-51-1/, accessed on 30 July 2026). Differentially expressed proteins with a *p*-value < 0.05 and a fold change ≥ 2 (compared to the control) were considered statistically significant.

### 2.12. Molecular Docking

To investigate the binding interaction between the outer membrane protein OmpF and ICAC, molecular docking was performed in this study [23]. The AlphaFold-predicted 3D structure of OmpF (https://alphafold.ebi.ac.uk/entry/P02931, accessed on 30 October 2024) and the molecular structure of ICAC were prepared using the “Prepared Protein” and “Prepared Ligands” modules in Discovery Studio 4.0, respectively. Molecular docking analysis was performed using the CDdock module of Discovery Studio 4.0, and binding conformation diagrams were generated using PyMOL 3.0.3. All docking parameters were kept at their default values. Subsequently, binding affinity and intermolecular interaction forces at the OmpF binding site were analyzed using the ligand interaction analysis module.

### 2.13. Animal Experiments

The in vivo animal experiment was performed based on a previously published protocol with minor modifications [24]. Specific pathogen-free (SPF) female BALB/c mice weighing 18 to 22 g were purchased from the Animal Experiment Center of Lanzhou Veterinary Research Institute, Chinese Academy of Agricultural Sciences. All animal procedures were approved by the Lanzhou Institute of Husbandry and Pharmaceutical Sciences, Chinese Academy of Agricultural Sciences [license No. 2024-019]. Mice were housed in individually ventilated cages (IVCs) under specific pathogen-free conditions at a constant temperature of 22 ± 2 °C, relative humidity of 50% ± 10%, and a 12 h light/dark cycle. All mice were provided with standard rodent chow and ad libitum access to sterile drinking water, and maintained on conventional bedding without additional environmental enrichment. All mice were randomly divided into four groups (*n* = 12 per group). Each mouse was intraperitoneally injected with the XJ-24 bacterial suspension (0.2 mL per 10 g body weight, approximately 3 × 10^8^ CFU/mL). One hour after infection, mice in each group were orally gavaged with PBS (control), ICAC (50 mg/kg), ciprofloxacin (10 mg/kg), and the combination of 50 mg/kg ICAC plus 10 mg/kg ciprofloxacin, respectively. Drug administration was repeated once every 24 h. After 7 days of continuous treatment, all mice were euthanized via cervical dislocation, and major organs were aseptically collected for subsequent analysis.

Bacterial load determination. Collected organ tissues were fully homogenized under aseptic conditions and serially diluted with sterile PBS. Each dilution was plated onto MacConkey agar and cultured at 37 °C for 24 h, followed by colony counting to determine tissue bacterial loads.

Histological evaluation. Sterile tissue samples, including liver, spleen, colon, and ileum, were fixed in 4% paraformaldehyde and embedded in paraffin. After H&E staining, tissue sections were observed and photographed under a light microscope for histopathological analysis. 

Real-time quantitative PCR (qRT-PCR) was performed to detect the mRNA levels of inflammatory cytokines IL-6, IL-1β and TNF-α. Total RNA was extracted from tissue samples using the SteadyPure Universal RNA Extraction Kit (Accurate Biology, Changsha, Hunan, China). Subsequently, cDNA was reverse-transcribed using the SPARKscript II RT Plus kit (with gDNA Eraser) (SparkJade, Jinan, Shandong, China). qRT-PCR was carried out with 2 × SYBR Green qPCR Mix (with ROX) (SparkJade, China). The *GAPDH* gene was used as an internal control. Relative gene expression levels were analyzed using the 2^−ΔΔCt^ method. All primer sequences in this study are showed in Table 1.

### 2.14. Statistical Analysis

All quantitative data in this study are presented as the mean ± standard deviation (mean ± SD). Statistical analysis was performed using SPSS 22 software (IBM, Armonk, NY, USA). For comparisons among three or more groups, one-way analysis of variance (one-way ANOVA) was applied, followed by Tukey’s honestly significant difference (HSD) post hoc test for multiple pairwise comparisons. *p* < 0.05 was considered statistically significant. Survival rates were calculated and plotted as Kaplan–Meier survival curves.

## 3. Results

### 3.1. Antibacterial Activity

Against the previously isolated multidrug-resistant (MDR) *E. coli* strain XJ-24, the checkerboard assay demonstrated that ICAC exerted a significant synergistic antibacterial effect with ciprofloxacin (FICI = 0.375) (Table 2). A similar synergistic effect was consistently observed in the standard susceptible strain ATCC 25922. 

### 3.2. Time–Kill Analysis

**Figure 1:**
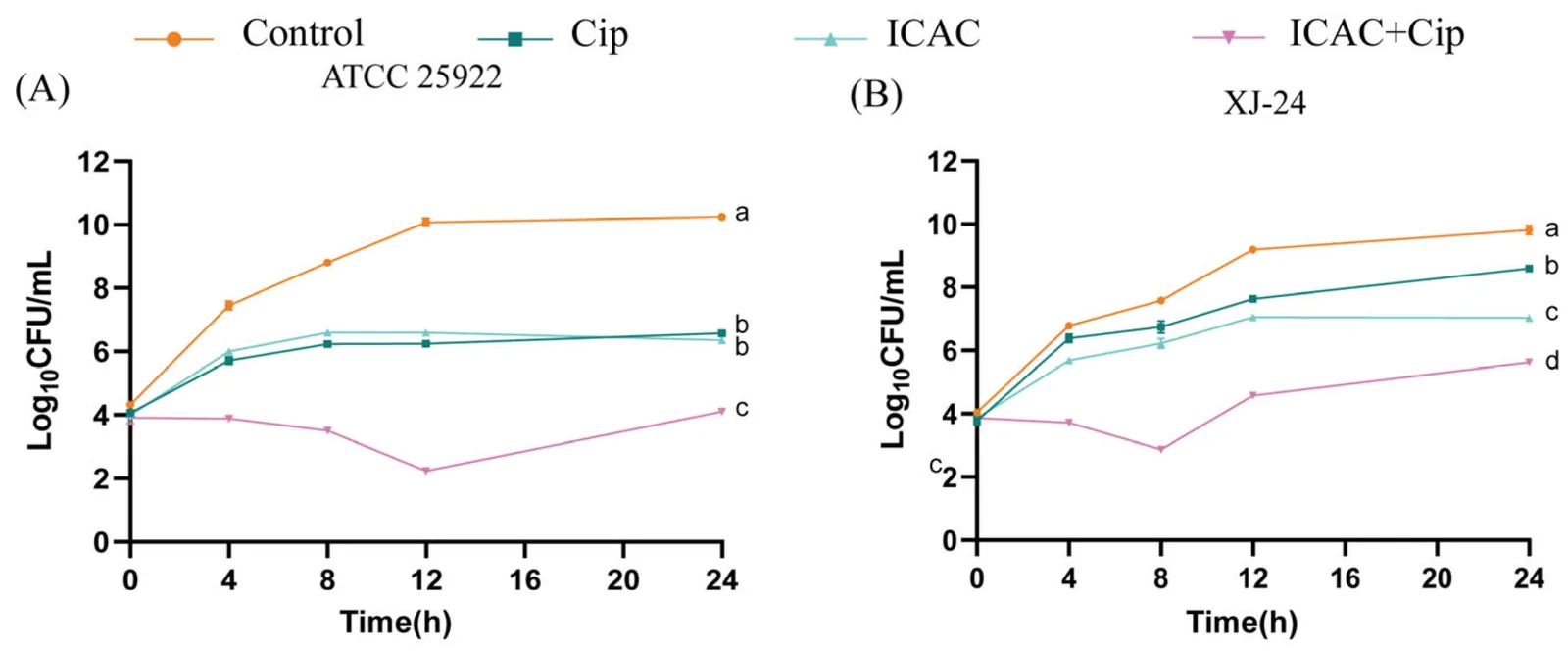
Antibacterial activity analysis. Time-dependent killing of ciprofloxacin and ICAC against *E. coli* ATCC 25922 (**A**) and XJ-24 (**B**). All experiments were performed three times and expressed as mean ± standard deviation. Different lowercase letters indicate significant differences among groups (*p* < 0.05, one-way ANOVA followed by Tukey’s HSD post hoc test). In panel (**A**), control (a) showed the highest bacterial count; Cip (b) and ICAC (b) were not significantly different from each other, but both significantly different from control (a); and ICAC + Cip (c) was significantly different from all other groups. In panel (**B**), control (a) showed the highest bacterial count and was significantly different from all other groups; Cip (b) was significantly different from control; ICAC (c) was significantly different from both control and Cip; and ICAC + Cip (d) was significantly different from all other groups.

To further evaluate the synergistic bactericidal activity of ICAC and ciprofloxacin, time–kill assays were performed against *E. coli* ATCC 25922 and XJ-24. As shown in Figure 1, the combination treatment exhibited significantly greater bactericidal effect than either agent alone against *E. coli* ATCC 25922. The combination of ICAC with ciprofloxacin reduced viable bacterial counts by 2-log_10_ and eradicated detectable bacteria at 12 h. For *E. coli* XJ-24, 8 h of combined treatment markedly reduced bacterial viability compared with the untreated control group. Notably, the combined regimen reduced viable bacterial counts by more than 10-fold relative to monotherapy groups. Collectively, these results demonstrated that the combination significantly enhanced the antibacterial efficacy of ICAC and ciprofloxacin.

### 3.3. Cell Wall Permeability Analysis

**Figure 2:**
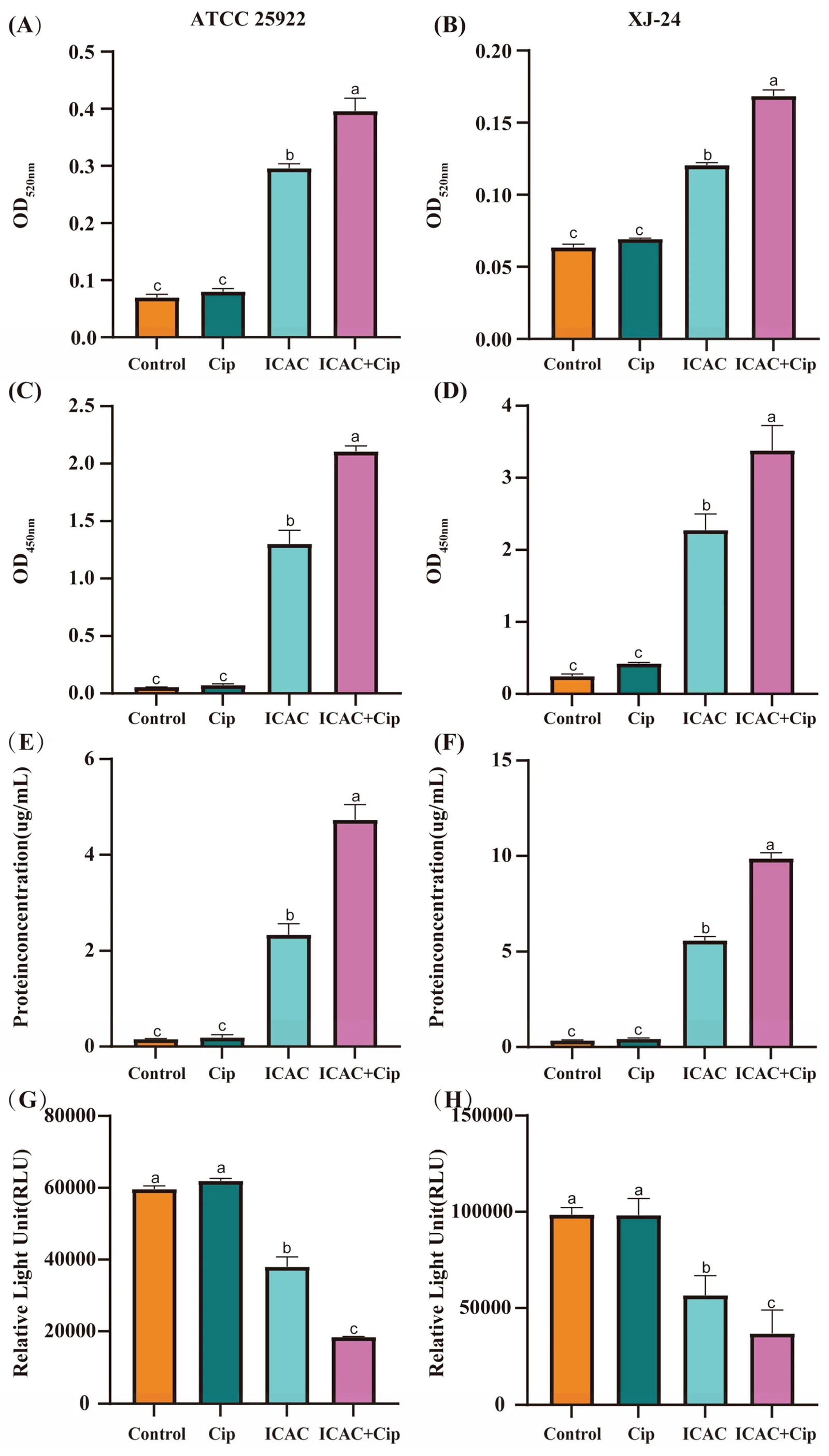
Effects of ICAC combined with ciprofloxacin on cell wall and membrane permeability. Contents of AKP (**A**,**B**), K^+^ (**C**,**D**), protein (**E**,**F**) and ATP (**G**,**H**) in the culture supernatant of *E. coli* ATCC 25922 and XJ-24 following treatment with ciprofloxacin and ICAC, either alone or in combination. All experiments were performed in triplicate, and data are expressed as mean ± standard deviation. Different lowercase letters indicate significant differences among groups (*p* < 0.05, one-way ANOVA followed by Tukey’s HSD post hoc test). Data are presented as mean ± SD. In panels (**A**–**F**), leakage of AKP (**A**,**B**), K^+^ (**C**,**D**) and protein (**E**,**F**) from *E. coli* ATCC 25922 and *E. coli* XJ-24 was lowest in the control (c), and there was no significant difference between the control (c) and Cip (c); ICAC (b) induced significantly greater leakage than both the control and Cip, while ICAC + Cip (a) differed significantly from all the other groups. In panels (**G**,**H**), ATP leakage from *E. coli* ATCC 25922 and *E. coli* XJ-24 was highest in the control (a) and Cip (a), and there was no significant difference between them; ICAC (b) induced significantly lower ATP leakage than both the control and Cip, while ICAC + Cip (c) showed the lowest ATP leakage and differed significantly from all the other groups.

The effect of ICAC on bacterial cell walls was determined by measuring extracellular AKP activity. Compared with the control group, significantly higher AKP activity was observed in the ICAC alone and combined treatment groups. Conversely, ciprofloxacin alone also enhanced AKP activity, but this change was not statistically significant (Figure 2A,B). These results demonstrated that ICAC disrupted the integrity of the *E. coli* cell wall and acted synergistically with ciprofloxacin to further increase extracellular AKP activity.

### 3.4. Inner Membrane (IM) Permeability

The ability of ICAC and ciprofloxacin, alone or in combination, to increase cell inner membrane permeability was assessed by measuring extracellular protein and K^+^ concentrations, as well as intracellular ATP levels. Combined treatment with ICAC and ciprofloxacin significantly elevated extracellular K^+^ (Figure 2C,D) and protein levels (Figure 2E,F), whereas intracellular ATP content was markedly reduced (Figure 2G,H); these effects were more pronounced than those in the control and monotherapy groups. Meanwhile, ciprofloxacin alone exerted no significant effect on bacterial inner membrane permeability. Collectively, these results indicated that ICAC disrupts inner membrane integrity, leading to leakage of intracellular contents.

### 3.5. IM Integrity Analysis

**Figure 3:**
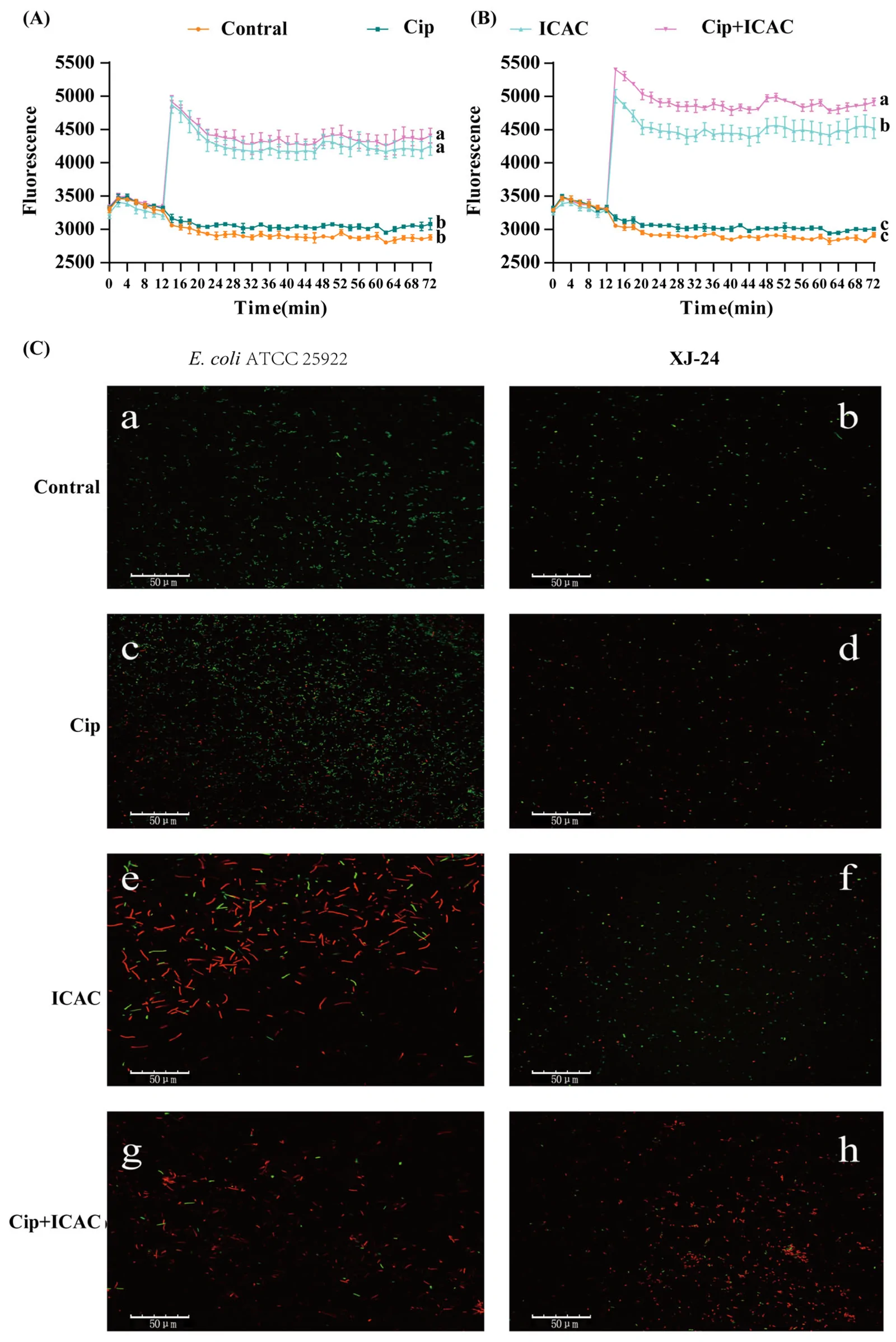
Effect of the ICAC–ciprofloxacin combination on bacterial inner membrane integrity. Quantified PI fluorescence intensity in *E. coli* ATCC 25922 (**A**) and XJ-24 (**B**) following treatment with ICAC and ciprofloxacin, alone or in combination. In panel (**A**), ICAC (a) and ICAC + Cip (a) showed the highest PI fluorescence intensity and were not significantly different from each other; control (b) and Cip (b) were not significantly different from each other, but were significantly lower than the ICAC and ICAC + Cip. In panel (**B**), ICAC + Cip (a) showed the highest PI fluorescence intensity and was significantly different from all other groups; ICAC (b) was significantly different from both control (c) and Cip (c); control (c) and Cip (c) were not significantly different from each other. (**C**) Representative CLSM micrographs (total magnification: 400×) of *E. coli* ATCC 25922 and XJ-24 under corresponding treatments. All experiments were performed in triplicate, and data are presented as mean ± standard deviation. Different lowercase letters indicate significant differences among groups (*p* < 0.05, one-way ANOVA followed by Tukey’s HSD post hoc test). Data are presented as mean ± SD.

cFDA and PI fluorescent dyes were used to evaluate bacterial inner membrane integrity. Severe membrane damage permits PI to penetrate bacterial cells and bind to genomic DNA, yielding red fluorescence. In contrast, cFDA freely diffuses into intact bacterial cells and was converted into carboxyfluorescein, which accumulates intracellularly and emits green fluorescence. Compared with the control and single ciprofloxacin-treated groups, the bacterial red fluorescence intensity was significantly elevated in the combination group, indicating that ICAC increases bacterial membrane permeability, thereby enhancing the susceptibility of *E. coli* ATCC 25922 (Figure 3A) and XJ-24 (Figure 3B) to ciprofloxacin. CLSM analysis further confirmed these observations. The red/green fluorescence ratio was markedly higher in the combined treatment group than in all other groups (Figure 3C). Notably, ATCC 25922 cells exhibited an elongated morphological phenotype after treatment with ICAC alone or the ICAC–ciprofloxacin combination. This morphological deformation may be attributed to ICAC-induced cell wall damage. In addition, no obvious morphological differences were observed between the ciprofloxacin-alone and control groups. Collectively, these findings demonstrated that ICAC triggers bacterial membrane damage in *E. coli*, and combination treatment further exacerbates membrane injury.

### 3.6. Cell Morphology Analysis

**Figure 4:**
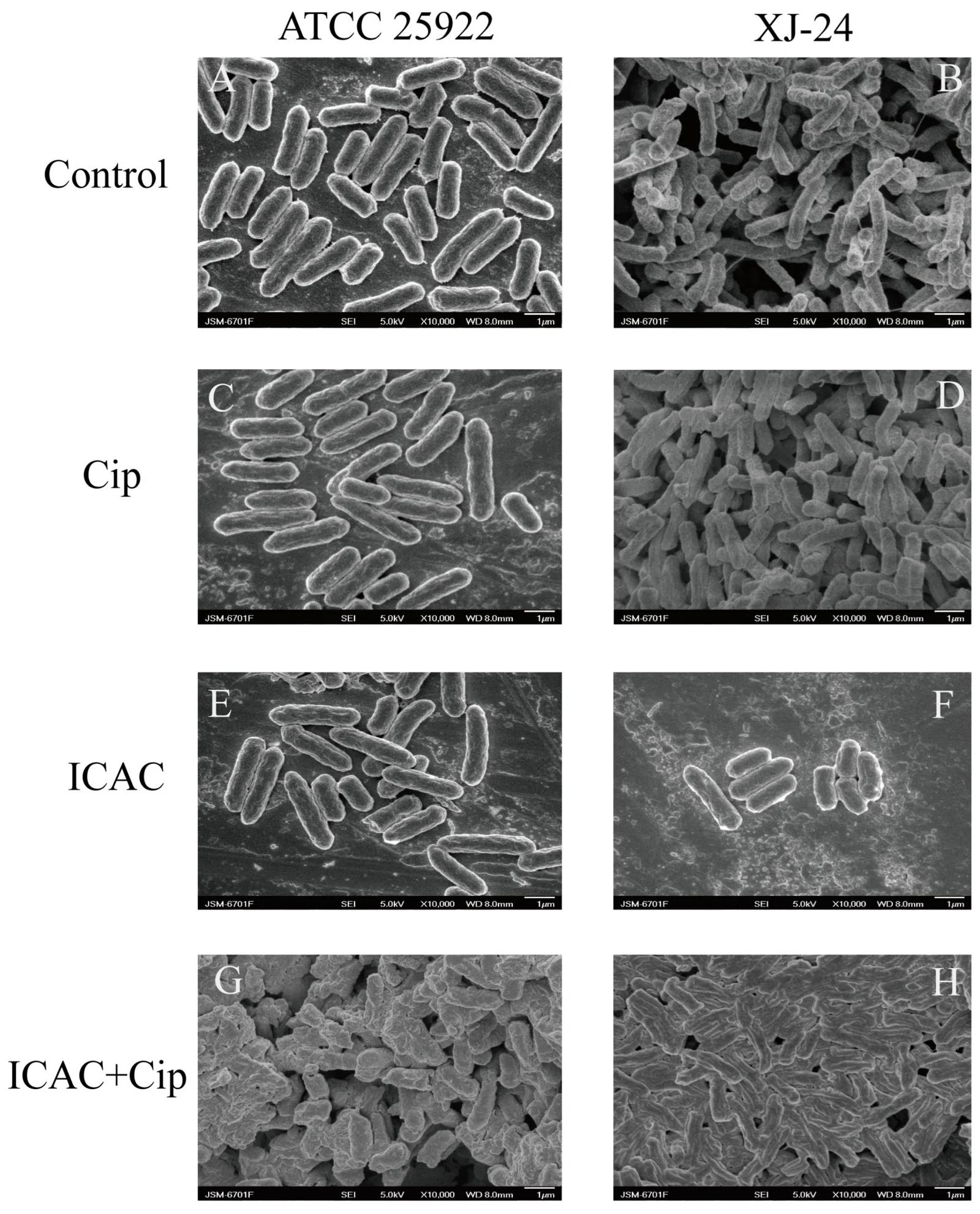
SEM image of *E. coli* ATCC 25922 and XJ-24 after treatment with control (**A**,**B**), Cip (**C**,**D**), ICAC (**E**,**F**), ICAC plus ciprofloxacin (**G**,**H**). Note that the panels are presented for qualitative comparison of cellular ultrastructure only; cell numbers are not equivalent across groups and do not reflect quantitative differences in viability.

To determine the damaging effects of ICAC and ciprofloxacin on bacterial membranes, scanning electron microscopy (SEM) was used to examine bacterial morphology and membrane integrity. As shown in Figure 4, untreated *E. coli* ATCC 25922 and XJ-24 exhibited smooth, intact cell surfaces. Following ICAC treatment alone, *E. coli* displayed rough surfaces accompanied by surface indentation and shrinkage. For cells receiving the ICAC–ciprofloxacin combination treatment, bacteria lost their characteristic rod-shaped morphology and exhibited severe lysis and cell aggregation. These observations suggested that the ICAC–ciprofloxacin co-treatment triggers bacterial membrane rupture, leading to leakage of intracellular contents, cell shrinkage, and eventual bacterial death. These morphological findings were consistent with the inner membrane (IM) permeability results described above.

### 3.7. DIA Quantitative Proteomic

**Figure 5:**
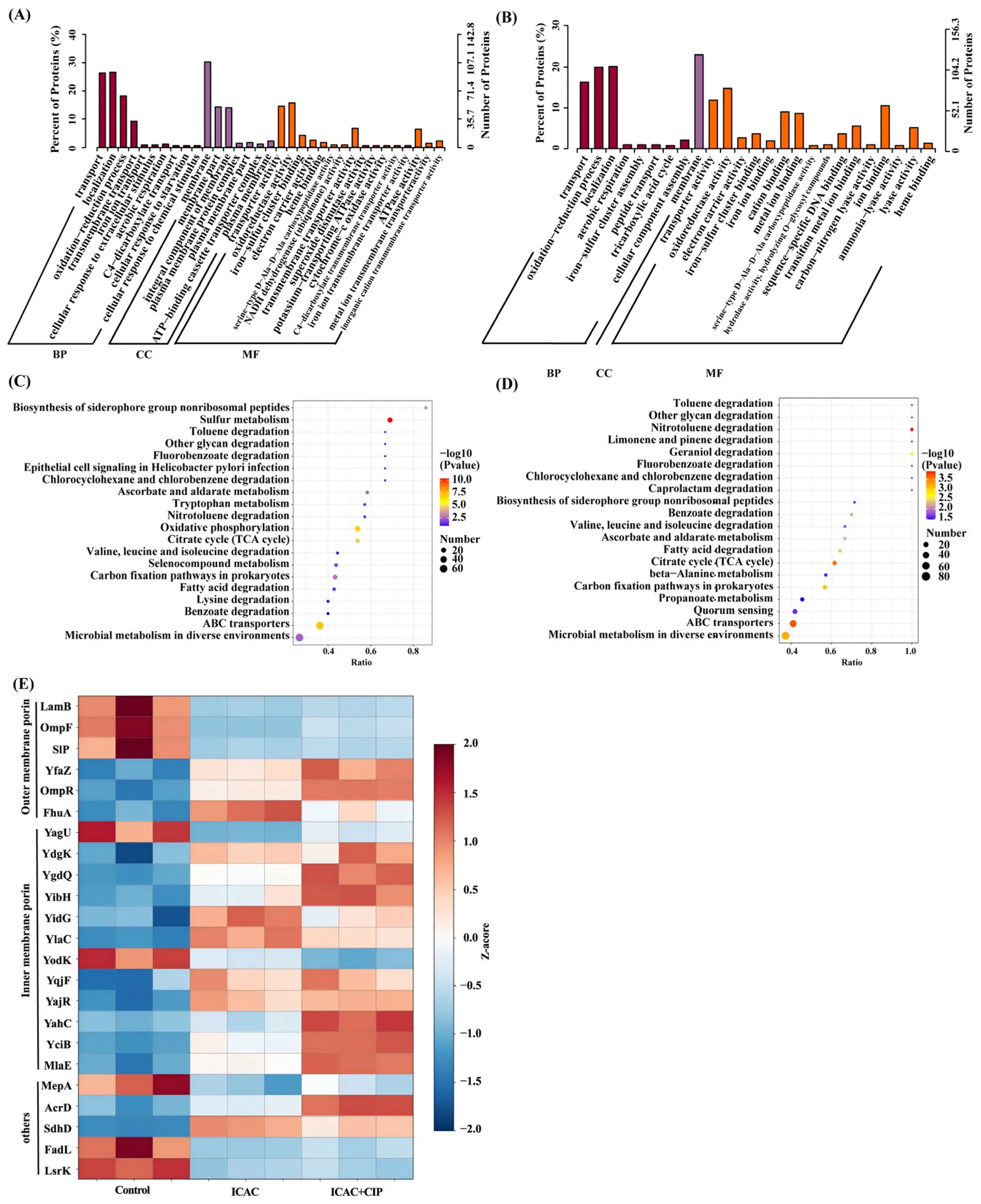
Quantitative proteomic analysis of *E. coli* following treatment with ICAC, ciprofloxacin (CIP), or their combination. GO functional annotation ((**A**) ICAC vs. control group, (**B**) ICAC vs. ICAC+ ciprofloxacin group) and KEGG enrichment analysis ((**C**) ICAC vs. control group, (**D**) ICAC vs. ICAC+ ciprofloxacin group) of differentially expressed proteins (DEPs) in *E. coli* following treatment with the combination of ICAC and ciprofloxacin. (**E**) Heatmap showing the relative expression levels (Z-score-normalized) of representative outer membrane and inner membrane proteins across the three treatment groups (control, ICAC, ICAC + CIP).

To further explore the underlying antimicrobial mechanism of ICAC, data-independent acquisition (DIA) quantitative proteomics was performed to identify proteomic changes in *E. coli*. A total of 3063 differentially expressed proteins were identified across the control, ICAC group, ciprofloxacin group, and combination treatment group. Comparative analysis revealed 896 differentially expressed proteins in the combination group relative to the ciprofloxacin-alone group, including 359 upregulated and 537 downregulated proteins. In contrast, the ICAC group exhibited 571 differentially expressed proteins, including 365 upregulated proteins and 206 downregulated proteins. Subsequently, Gene Ontology enrichment analysis was performed using the GO database (http://www.geneontology.org/, accessed on 22 February 2024) to functionally annotate these differentially expressed proteins. As shown in Figure 5A,B, comparison between the combination and ciprofloxacin-alone groups showed that the most enriched molecular function (MF) terms were oxidoreductase activity and transporter activity. For the cellular component (CC) category, membrane-associated proteins constituted the predominant enriched fraction. In the biological process (BP) category, the most highly enriched terms were transport and protein localization. For the ICAC group, GO enrichment results demonstrated that the top enriched BP terms were localization and transport; the dominant CC term was membrane component; and the primary MF terms were oxidoreductase activity and transporter activity.

Moreover, KEGG pathway enrichment analysis was performed for the ICAC group and the combined treatment group (Figure 5C,D). Several significantly enriched pathways, including ABC transporters, citrate cycle (TCA cycle), and fatty acid degradation, were identified in both the ICAC and combination treatment groups. 

To further characterize the effects of ICAC on bacterial membrane systems, we performed an integrated analysis of all differentially expressed membrane-associated proteins, and their relative expression profiles are presented as a Z-score-normalized heatmap (Figure 5E). The heatmap displays expression patterns across the control, ICAC monotherapy, and combination treatment groups, with proteins grouped by subcellular localization (outer membrane, inner membrane, and membrane-associated). Consistent with the GO enrichment results, we observed widespread dysregulation of membrane proteins in both treatment groups. Notably, the outer membrane protein OmpF exhibited pronounced downregulation, with a log_2_ fold change of −4.19 in the ICAC group and −2.86 in the combination treatment group relative to the untreated control. As a well-characterized major uptake channel for fluoroquinolone antibiotics including ciprofloxacin, dysregulation of OmpF is directly linked to bacterial antibiotic susceptibility. This observation suggests that ICAC disrupts outer membrane barrier function, and OmpF represents a potential molecular target of ICAC. In addition, multiple inner membrane ABC transporters and energy metabolism-associated membrane proteins were significantly downregulated, implying impaired nutrient transport and energy generation. These proteomic changes provide a direct molecular basis for the increased membrane permeability observed in our phenotypic assays, including enhanced AKP leakage, K^+^ and protein efflux, reduced intracellular ATP levels, and increased PI staining intensity. Collectively, these findings demonstrate that ICAC may target OmpF, disrupting bacterial membrane homeostasis by modulating the expression of key membrane proteins, which synergizes with ciprofloxacin to exert enhanced antibacterial activity.

### 3.8. Molecular Docking

**Figure 6:**
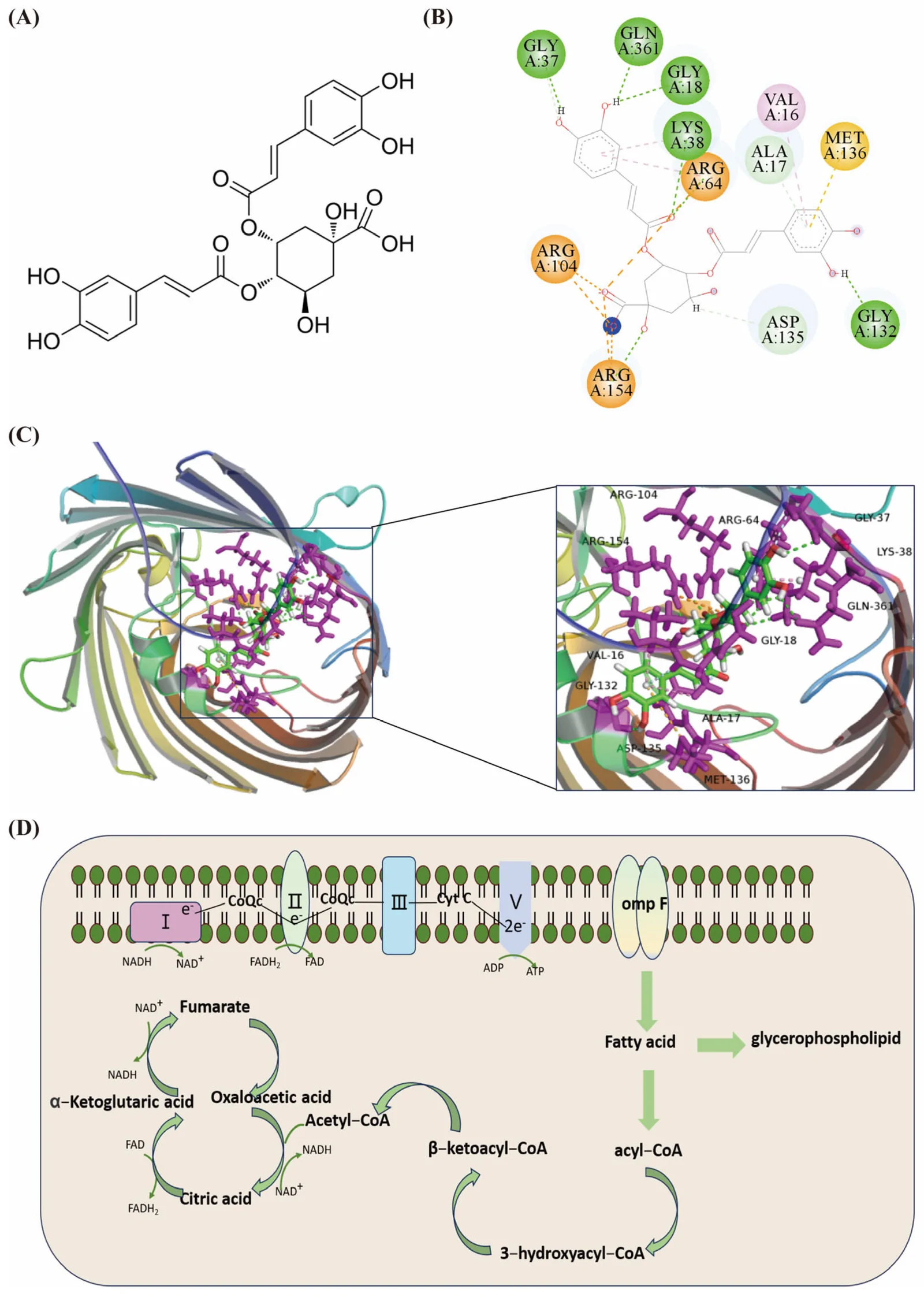
The interaction between ICAC and OmpF was simulated by molecular docking. (**A**) Chemical structure of ICAC. (**B**) The 2D interaction pattern between OmpF and ICAC. (**C**) Spatial conformation of ICAC and its interacting amino acid residues in the OmpF binding pocket. (**D**) Proposed inhibitory mechanism of ICAC against *E. coli*.

Molecular docking simulations were conducted to predict the putative binding mode and binding affinity of ICAC to OmpF. As shown in Figure 6A–C, five amino acid residues (Gly 37, Gly 18, Gln 361, Lys 38 and Gly 132) formed hydrogen bonds with the ICAC ligand. ICAC formed carbon–hydrogen bonds with Ala 17 and Asp 135, and salt bridges with Arg 64, Arg 104, and Arg 154. The binding energy of the OmpF-ICAC complex was −62.163 kcal/mol, reflecting favorable binding affinity. 

### 3.9. ICAC Enhances the Therapeutic Efficacy of Ciprofloxacin In Vivo

**Figure 7:**
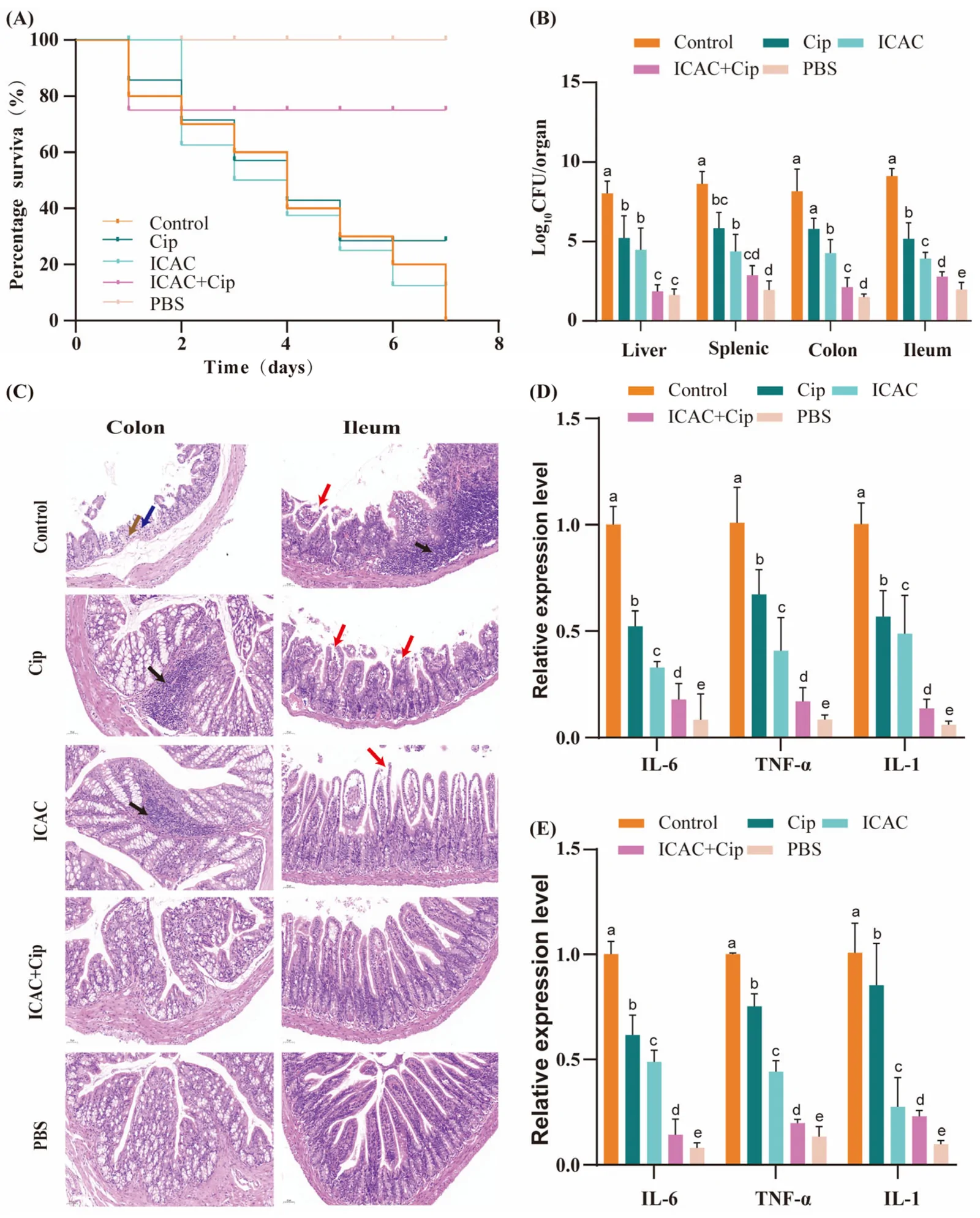
ICAC potentiates the antibacterial efficacy of ciprofloxacin in vivo. (**A**) Survival of mice treated with ICAC or ciprofloxacin alone, or in combination. (**B**) Bacterial load in different organs of mice following treatment with ICAC and ciprofloxacin, either alone or in combination (n = 12 per group). In the liver, control (a) showed the highest bacterial load and was significantly different from all other groups; Cip (b) and ICAC (b) were not significantly different from each other but were significantly higher than the ICAC + Cip (c) and PBS (c); ICAC + Cip (c) and PBS (c) were not significantly different from each other. In the spleen, control (a) showed the highest bacterial load and was significantly different from all other groups; Cip (bc) and ICAC (b) were not significantly different from each other; ICAC + Cip (cd) and PBS (d) were not significantly different from each other, and Cip (bc) was not significantly different from ICAC + Cip (cd); ICAC (b) was significantly different from ICAC + Cip (cd) and PBS (d), and PBS (d) showed the lowest bacterial load. In the colon, control (a) and Cip (a) showed the highest bacterial loads and were not significantly different from each other; ICAC (b), ICAC + Cip (c) and PBS (d) were significantly different from all other groups. In the ileum, control (a) showed the highest bacterial load and was significantly different from all other groups, followed by Cip (b), ICAC (c) and ICAC + Cip (d); PBS (e) showed the lowest bacterial load, with significant differences among all groups. (**C**) Morphological changes in various organs in different treatment groups evaluated by H&E staining. Hyperplasia of connective tissue, blue arrow; shedding of epithelial cells in the mucosal layer, red arrow; lymphocyte aggregation, black arrow; lymphocytes infiltrate, brown arrow. (**D**) Relative mRNA expression levels of *IL-6*, *TNF-α* and *IL-1β* in ileum of mice from different treatment groups (n = 12 per group). In the ileum, for *IL-6*, *TNF-α* and *IL-1β*, control (a) showed the highest expression levels and was significantly different from all other groups, followed by Cip (b), ICAC (c) and ICAC + Cip (d); PBS (e) showed the lowest expression levels, with significant differences among all groups. (**E**) Relative mRNA expression levels of *IL-6*, *TNF-α* and *IL-1β* in colon of mice from different treatment groups (n = 12 per group). In the colon, for *IL-6*, *TNF-α* and *IL-1β*, control (a) showed the highest expression levels and was significantly different from all other groups, followed by Cip (b), ICAC (c) and ICAC + Cip (d); PBS (e) showed the lowest expression levels, with significant differences among all groups. Different lowercase letters indicate significant differences among groups (*p* < 0.05, one-way ANOVA followed by Tukey’s HSD post hoc test). Data are presented as mean ± SD.

Having confirmed the in vitro synergistic activity of ICAC and ciprofloxacin via FICI calculation and time–kill assay, we further validated the in vivo therapeutic efficacy of this combination therapy using an intraperitoneal infection mouse model. Accordingly, an intraperitoneal infection mouse model was constructed to evaluate its synergistic effect in vivo. As presented in Figure 7A, the survival rate of mice receiving ICAC and ciprofloxacin combination therapy reached 83.33%, which was markedly higher than that of the ciprofloxacin group (58.33%) and ICAC group (41.67%). Furthermore, bacterial burdens were quantified in the liver, spleen, colon and ileum. As shown in Figure 7B, the combination treatment group exhibited significantly lower CFU counts than the monotherapy groups. In addition, H&E staining showed that mice from the ICAC-alone and combination treatment groups displayed alleviated pathological lesions relative to other experimental groups (Figure 7C). Finally, compared to the control group, the mRNA expression levels of pro-inflammatory cytokines IL-6, IL-1β, and TNF-α were significantly reduced in the ileum (Figure 7D) and colon (Figure 7E) of mice in the combination treatment group.

## 4. Discussion

Pathogenic *E. coli* is a common Gram-negative bacterium that can cause severe diarrhea and urinary tract infections in animals and humans, and even endanger human and animal life in cases of infection with virulent strains [25]. In recent years, with the emergence of MDR bacteria, antibiotic resistance has become increasingly serious, posing substantial challenges to clinical treatment [26]. Therefore, strategies to restore the antibacterial efficacy of antibiotics and reduce the required therapeutic doses are urgently required. Recent studies have shown that combination therapy with non-antibiotic agents represents a promising strategy to enhance the effectiveness of existing antibiotics [27]. Compared with antibiotic monotherapy, drug combinations can lower the effective antibiotic concentration, thereby alleviating in vivo drug-related toxic side effects and improving therapeutic outcomes [28]. Accordingly, the combined administration of such compounds constitutes a promising therapeutic approach to overcome bacterial resistance.

ICAC is a phenolic acid compound with antioxidant, anti-inflammatory, antibacterial, and other pharmacological activities [29,30]. Nevertheless, few previous studies have explored the synergistic bactericidal effects of ICAC combined with conventional antibiotics. In this study, we demonstrated that ICAC effectively reduces the therapeutic dose of ciprofloxacin against multidrug-resistant *E. coli*, and that the combination of ICAC and ciprofloxacin exerts a significant synergistic antibacterial effect. Mechanistically, this combination treatment achieves potent antibacterial activity by disrupting the structural integrity of the cell wall as well as the inner and outer membranes of *E. coli*. Collectively, our findings suggest that ICAC is a promising antibacterial synergist candidate for the treatment of MDR bacteria.

In vitro antibacterial assays demonstrated that ICAC effectively enhances bacterial susceptibility to multiple antibiotics, and the combination of ICAC and ciprofloxacin exerted a significant synergistic antibacterial effect against *E. coli*. This synergistic bactericidal phenotype was further confirmed by time–kill assay. Analysis of bacterial colony counts for *E. coli* ATCC 25922 and XJ-24 at various incubation time points confirmed the robust bactericidal activity of the combined regimen. In contrast, the antibacterial efficacy of single-drug treatments was markedly attenuated after 4 h of incubation.

Plant-derived compounds exert antibacterial activity by disrupting the structural integrity of bacterial cell walls and membranes. For example, baicalein acts synergistically with doxycycline to damage membrane integrity via direct binding to bacterial phospholipids [17]. Consistent with these findings, scanning electron microscopy (SEM) observations confirmed similar bacterial morphological damage. Following combined treatment of ICAC and ciprofloxacin, *E. coli* exhibited remarkable morphological and structural alterations, including cell collapse, shrinkage, and leakage of intracellular contents. Building on these insights, we speculate that ICAC enhances the bactericidal performance of ciprofloxacin by disrupting bacterial morphology and membrane structure, thereby exerting a synergistic antibacterial effect.

In addition, damage to cell wall and membrane integrity can be quantitatively verified by detecting the extracellular release of ATP, K^+^, AKP, and total proteins from bacterial culture supernatants. AKP is located in the periplasmic space between the bacterial cell wall and cell membrane. Structural damage to the cell wall allows AKP to leak into the extracellular environment, and extracellular AKP activity can therefore serve as a reliable biomarker for evaluating cell wall integrity [31]. In this study, the combined treatment group exhibited significantly higher extracellular AKP levels than the single-drug groups, which was consistent with previous mechanistic studies. Similarly, markedly increased extracellular K^+^ and protein concentrations and decreased intracellular ATP levels were observed in the combination group compared with monotherapy groups. These findings further confirmed that the combination of ICAC and ciprofloxacin compromised the bacterial membrane structure of *E. coli*, triggering the leakage of intracellular components. Consistent with these phenotypic changes, CLSM detection based on PI penetration further validated the severe membrane damage induced by combination treatment. 

Quantitative proteomic analysis demonstrated that among all detected bacterial membrane proteins, OmpF exhibited the most pronounced differential expression in both the ICAC monotherapy and combination treatment groups. In addition, molecular docking simulation verified the high binding affinity of ICAC toward OmpF, with stable interactions formed between ICAC and key amino acid residues of OmpF. Previous studies have demonstrated that upregulated OmpF expression inhibits fatty acid biosynthesis [32]. Fatty acids are essential for synthesizing glycerophospholipids, which are the fundamental structural components of bacterial cell membranes. Bacterial structural integrity is tightly dependent on energy metabolism, and insufficient energy supply inevitably impairs membrane stability. Additionally, fatty acid β-oxidation serves as the primary pathway for fatty acid degradation. Reduced fatty acid availability inhibits β-oxidation, decreases acetyl-CoA production, and further blocks tricarboxylic acid (TCA) cycle progression, ultimately disrupting bacterial energy metabolism. Collectively, these results suggest that ICAC may target OmpF, thereby inhibiting fatty acid degradation and energy metabolism, which in turn impairs membrane synthesis and increases bacterial membrane permeability (Figure 6D).

Given the excellent in vitro synergistic antibacterial activity of the ICAC–ciprofloxacin combination, we further validated its in vivo efficacy using a mouse intraperitoneal infection model. In vivo results demonstrated that combination treatment significantly improved mouse survival rates, reduced organ bacterial loads, and suppressed the expression of pro-inflammatory cytokines (IL-6, TNF-α, and IL-1β). In summary, ICAC synergizes with ciprofloxacin in vitro by disrupting bacterial cell wall and membrane integrity, increasing membrane permeability, and interfering with bacterial energy metabolism. This study demonstrated the formally verified in vitro synergistic antibacterial activity of ICAC combined with ciprofloxacin (via FICI and time–kill assays), as well as the enhanced in vivo therapeutic efficacy of this combination regimen in a mouse peritonitis model. ICAC acts as a promising antibacterial adjuvant for ciprofloxacin, which mitigates antibiotic resistance pressure, reduces drug-associated toxic side effects, and maintains stable therapeutic efficacy. Therefore, the combination of ICAC and ciprofloxacin represents a novel and potent therapeutic strategy for the treatment of infectious diseases caused by the tested pathogenic *E. coli*. 

Nevertheless, this study still has several limitations. First, synergistic activity of ICAC combined with ciprofloxacin was characterized using only one MDR *E. coli* isolate (XJ-24) and the reference susceptible strain ATCC 25922. Therefore, we cannot conclude that such synergism holds universally for all drug-resistant *E. coli*; more independent clinical multidrug-resistant *E. coli* isolates with diverse resistance backgrounds are needed to verify the generalizability of this combination. Second, the proposed target OmpF was inferred mainly from quantitative proteomic profiling and molecular docking simulation in the present work. Direct functional validation, including construction of ompF-knockout, complementation and overexpression strains, together with biophysical assays such as thermal-shift assay, remains absent. Thus, the causal relationship between ICAC-OmpF interaction and the downstream antibacterial phenotypes needs further experimental confirmation. Third, only a single mouse intraperitoneal infection model and a single drug-resistant *E. coli* strain were used for in vivo efficacy validation, and no formal in vivo synergy analysis was performed in the current study. Fourth, in vivo therapeutic evaluation targeting livestock and poultry was not performed. Fifth, the long-term toxicological safety of ICAC remains uncharacterized. Future studies will focus on expanding animal models and evaluating long-term biosafety to further support the clinical application potential of this combination strategy.

## 5. Conclusions

The combination of ICAC and ciprofloxacin disrupts the cell wall and membrane integrity, potentially via targeting the outer membrane protein OmpF, and exerts synergistic antibacterial activity against the tested *E. coli* strains through multiple regulatory pathways. This combinatorial regimen also achieves effective therapeutic outcomes in a mouse peritonitis model, providing a credible theoretical basis for the development of novel antibacterial adjuvants.

## Figures and Tables

**Table 1:** Primer sequence for qPT-PCR.

Gene	Primer Sequence
*IL-6*	F-CCGGAGAGGAGACTTCACAG
	R-CAGAATTGCCATTGCACAAC
*TNF-α*	F-ACCCCATGGATCTCAAAGAC
	R-GTGGGTGAGGAGCACGTAGT
*IL-1β*	F-GCTGCTTCCAAACCTTTGAC
	R-AGCTTCTCCACAGCCACAAT
*GAPDH*	F-AGGTCGGTGTGAACGGATTTG
	R-CCAGTTGGTAACAATGCCATGT

**Table 2:** The MIC (μg/mL) and FICI values of ICAC combined with different antibiotics in *E. coli* ATCC 25922 and XJ-24.

Antibiotic	MIC		FICI	
	ATCC 25922	XJ-24	ATCC 25922	XJ-24
Ceftriaxone Sodium	0.125(S)	16(R)	0.5625	0.75
Polymyxin	0.5(S)	256(R)	0.5625	0.625
Tigecycline	0.25(S)	32(R)	0.5625	0.75
Meropenem	0.03125(S)	0.015625(S)	1.5	1.25
Amikacin	4(S)	64(R)	0.625	0.375
Kanamycin	0.25(S)	64(R)	0.75	0.625
Aztreonam	0.25(S)	2(S)	0.5	1.25
Tetracycline	0.5(S)	64(R)	0.375	0.5625
Ciprofloxacin	0.0125(S)	64(R)	0.28125	0.375
Amoxicillin	4(S)	32(R)	1.25	0.75

FICI ≤ 0.5, synergism; 0.5 < FICI ≤ 1.0, additive effect; 1.0 < FICI ≤ 2.0, no effect; FICI > 2, antagonistic effect.

## Data Availability

The original contributions presented in this study are included in the article. Further inquiries can be directed to the corresponding authors.
